# Efficacy of heat-killed *Lactococcus lactis* JCM 5805 on immunity and fatigue during consecutive high intensity exercise in male athletes: a randomized, placebo-controlled, double-blinded trial

**DOI:** 10.1186/s12970-018-0244-9

**Published:** 2018-08-02

**Authors:** Yuta Komano, Kazunori Shimada, Hisashi Naito, Kosuke Fukao, Yoshihiko Ishihara, Toshio Fujii, Takeshi Kokubo, Hiroyuki Daida

**Affiliations:** 1 Department of Cardiovascular Medicine, Juntendo University Graduate School of Medicine, Bunkyo-ku, Tokyo, Japan; 2 Graduate School of Health and Sports Science, Juntendo University, Inzai, Chiba, Japan; 3 Department of humanities and Social Sciences, School of Science and Technology for Future Life, Tokyo Denki University, Adachi-ku, Tokyo, Japan; 4 Research Laboratories for Health Science & Food Technologies, Kirin Co., Ltd., Yokohama, Kanagawa Japan

**Keywords:** Dendritic cells, High intensity exercise, LC-plasma, Upper respiratory tract infections, Fatigue

## Abstract

**Background:**

*Lactococcus lactis* JCM 5805 (LC-Plasma) is a unique lactic acid bacteria (LAB) which activates plasmacytoid dendritic cells (pDC). We aimed to evaluate the effect of LC-Plasma on dendritic cell (DC) activity and subjective indices of upper respiratory tract infections (URTI) and fatigue in athletes under high intensity exercise.

**Methods:**

We conducted a randomized, placebo-controlled, double-blinded trial. Fifty-one male subjects belonging to a university sports club were randomized into placebo (*n* = 25) and LC-Plasma (*n* = 26) groups. Individuals ingested placebo capsules containing cornstarch or LC-Plasma capsules containing 100 billion cells of heat-killed LC-Plasma per day for 13 days. During the intervention period, subjects performed high intensity exercise according to their sports club training regime. Blood and saliva sampling were obtained at days 1 and 14, and physical conditions were recorded in a diary. We investigated expression of maturation markers on DCs, muscle damage and stress markers and used student’s *t* test adjusted by Bonferoni’s method for multiple comparison between groups. These data were presented as mean ± SD. We also investigated cumulative days of symptoms regarding infections and fatigue and used Chi-square test for comparison between groups. These data were presented as cumulative number.

**Results:**

CD86 as maturation marker on pDC was significantly increased in the LC-Plasma group at day 14 (Placebo: 296 ± 70 vs. LC-Plasma: 365 ± 115; Mean Fluorescent Intensity; *p* = 0.013). Cumulative days of URTI were significantly lower in the LC-Plasma group (Placebo: URTI positive 56, URTI negative 256 vs. LC-Plasma: URTI positive 39, URTI negative 299; days; *p* = 0.028) and symptoms like sneeze or running nose were significantly lower in the LC-Plasma group (Placebo: Symptom positive 52, Symptom negative 258, vs. LC-Plasma: Symptom positive 36, Symptom negative 301; days; *p* = 0.032). Moreover, the cumulative days of fatigue were significantly fewer in the LC-Plasma group (Placebo: Symptom positive 128, Symptom negative 182, vs. LC-Plasma: Symptom positive 110, Symptom negative 225; days; *p* = 0.032). Markers of muscle damage and stress markers were not significantly different between groups.

**Conclusion:**

We consider that heat-killed LC-Plasma supplementation relieves morbidity and symptoms of URTI via activation of pDC and decreases fatigue accumulation during consecutive high intensity exercise in athletes. However, LC-Plasma ingestion did not affect markers of muscle damage and stress.

**Trial registration:**

UMIN-CTR, UMIN000020372. Registered 28 December 2015.

## Background

It has been well-known that prolonged high intensity exercise (HIE) increases the risk of upper respiratory tract infections (URTI). URTI are common and account for about 65% of non-injury-related symptoms in athletes [1, 2]. It is also known that the increase of URTI by HIE is attributed to a decrease in immunity [3]. Several reports demonstrated that the concentration of salivary secretary immunoglobulin A (sIgA) and natural killer cells, well-known immunocytes against viral and bacterial infections, decreased after prolonged HIE [4–6]. It is understood that immunity and fatigue are closely related, and that chronic fatigue patients are susceptible to virus infections [7]. Therefore, for athletes, not only decrease of immunity but also increase of fatigue is a serious problem.

Dendritic cells (DCs) play pivotal roles in the immune system such as antigen-presentation [8]. DCs are classified into plasmacytoid DC (pDC) and myeloid DC (mDC) based on phenotype and function [9]. pDC are responsible for the antiviral response. When viral infections are detected by pDC via toll-like receptor 7 (TLR7) or TLR9, pDC produce interferon-α (IFN-α) [10] and induce expression of antiviral factors for inhibition of viral replication and spread [11]. It was reported that 70% of URTI morbidity is caused by viral infections [12]. mDC are responsible for the response to bacterial infections, and are activated by bacteria ligands via TLR1, TLR2, and TLR6 [9]. mDC produce IL-10 and IL-12 [13, 14]. It was reported that 70% of URTI morbidity is caused by viral infections [12]. Therefore, the study of pDC is more important to improve URTI morbidity. There are a few reports studying the relationship between pDC and single HIE although there is no report regarding changes of pDC function after consecutive HIE [15, 16].

Probiotics such as lactic acid bacteria (LAB) are well-known food supplements to improve immune function and decrease URTI symptoms [17–19]. *Lactobacillus plantarum*, *Lactobacillus paracasei Lactobacillus gasseri, Bifidobacterium longum*, *Bifidobacterium bifidum* and *Lactobacillus acidophilus* supplementation were reported to decrease the risk of URTI in general healthy subjects. It was also reported in athletes that *Lactobacillus salivarius* improved sIgA concentration [20, 21].

*Lactococcus lactis* JCM 5805 (LC-Plasma, also referred to as *Lactococcus lactis* strain plasma) has been shown to be a unique LAB which activates pDC in vitro and in vivo [22]. There are several reports regarding the efficacy of LC-Plasma supplementation in healthy subjects. LC-Plasma yogurt intake for 4 weeks activated maturation markers of pDC and intake for 12 weeks reduced the cumulative number of incidence days of influenza-like symptoms [23, 24]. In addition, heat-killed LC-Plasma supplementation for 12 weeks reduced the number of incidence days of symptoms related to URTI [25]. However, there is no report regarding the efficacy of LC-Plasma supplementation on pDC activation in athletes under HIE. Moreover, the influence of LC-Plasma intake on fatigue in athletes has never been evaluated. Based on the fact that pDCs are affected by single HIE and that LC-Plasma activates pDC and improves URTI, we hypothesized that ingestion of LC-Plasma would maintain pDC activity and suppress infection morbidity even during consecutive HIE. Moreover, we also hypothesize that LC-Plasma intake is effective for fatigue accumulation of athletes. To this end, we conducted a randomized, placebo-controlled, double-blinded trial to examine the effects of heat-killed LC-Plasma supplementation on maturation markers on DCs, and subjective indices correlating infections and fatigue under HIE. The primary efficacy outcomes were maturation markers (CD86, HLA-DR) on DCs and subjective indices (evaluation of influenza and URTI, and symptom severity including fatigue). Secondary outcomes were markers of muscle damage (creatine phospho kinase (CPK) and lactate dehydrogenase (LDH)) and stress markers (adrenaline and salivary cortisol).

## Methods

### Subjects

**Fig. 1:**
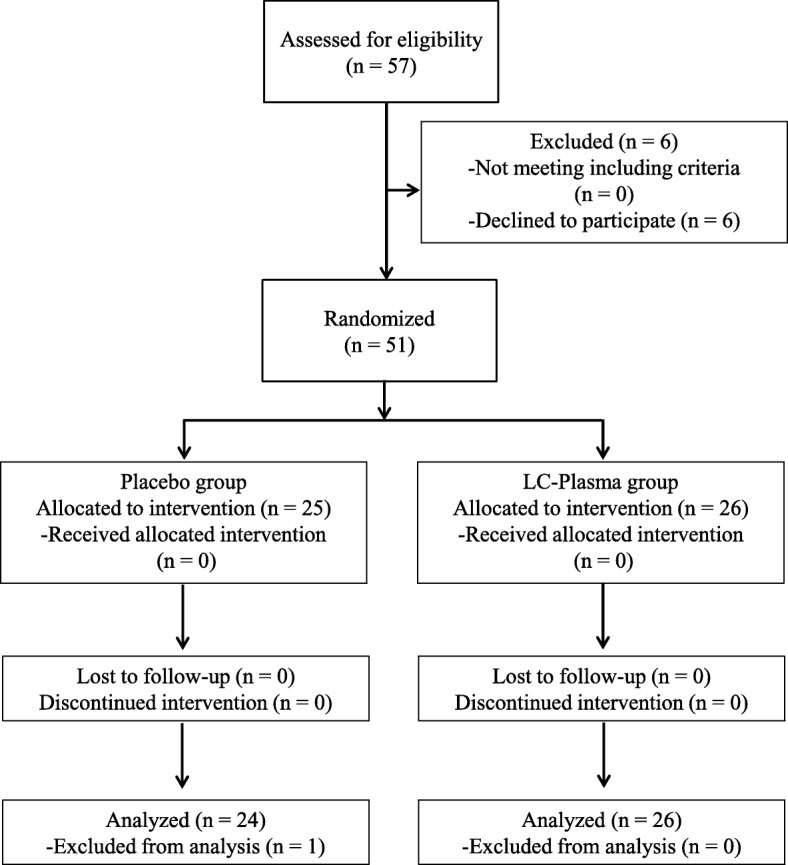
Consolidated standards of trials diagram: enrollment, random, assignment, and follow-up of subjects

The Consolidated Standards of Trials diagram for this study is shown in Fig. 1. The required sample size was estimated using data of a previous clinical intervention study regarding LC-Plasma [23]. Based on previous data of maturation markers on pDC, more than 20 subjects in each group would allow detection of differences between groups with a 5% statistical significant level. Initially, 57 healthy male athletes over 20 years of age were recruited between December 2015 and January 2016 from students belonging to sports clubs (track and field, futsal, and football) of the Juntendo University. Informed consent was obtained from 51 subjects (6 subjects declined to participate). No one met the exclusion criteria (subjects with severe chronic disease, steroid treatment, previous history of high risk for exercise, under treatment for pollinosis, and positive against HBV antigen, HCV antibodies, HIV antibodies, or HTLV-1 antibodies, and who could not stop eating functional foods or supplements containing lactic acid bacteria, oligosaccharide and fermented foods). A total of 51 subjects were randomly allocated to the placebo (*n* = 25) or LC-Plasma (*n* = 26) groups using a stratified randomization method. No participants dropped out during the intervention period. One subject in the placebo group was excluded from the analysis because of ingestion of steroid drugs included in the exclusion criteria during the intervention period. We analyzed data from 24 subjects in the placebo group and 26 subjects in the LC-Plasma group.

### Study design

A randomized, double blind, placebo-controlled trial was conducted with two groups. A stratified randomization method was used to balance the number subjects in the groups. Subjects were randomly assigned to the placebo group or the LC-Plasma group in a 1:1 ratio. Placebo capsules contained cornstarch (Showa Sangyo Co., Ltd., Tokyo, Japan) and LC-Plasma capsules contained approximately 100 billion cells of heat-killed dried powder of LC-Plasma (KYOWA HAKKO BIO CO., Ltd., Tokyo, Japan) and cornstarch. Each subject ingested 1 capsule per day for 13 days between January and February 2016. Neither the study team nor the subjects could distinguish between the placebo and the LC-Plasma capsules, and the capsule codes were stored by a responsible person of allocation until all analyses were finished and the codes were opened after the data set was locked. During the intervention period, subjects exercised according to the training regime of their sports clubs. Subjects filled the records of capsule intake, exercise, meals, and a physical condition questionnaire in the diary every day. We adopted the amount of physical activity as metabolic rate-hour (MET-h) which is globally used index based energy consumption [26]. We calculated MET-h from records of exercise according to a previous report [27].

### Blood and saliva sampling

Blood and saliva samples were collected twice, before and after the intervention period (day 1 and day 14) and at the same time of the day (between 08:00 to 09:00 a.m.). Saliva samples were collected using salimetrics oral swabs (Salimetrics LLC, PA, USA) according to the manufacturer’s instructions.

### Measurement of blood and saliva samples

CPK and LDH were analyzed using the Japan Society of Clinical Chemistry recommended methods by the SRL, Inc. (Tokyo, Japan). Serum adrenaline concentration was measured using an ELISA kit (Arigo Biolaboratories Corp., Hsinchu City, Taiwan). Salivary cortisol was measured by an immunoassay kit according to the manufacturer’s instructions (Salimetrics LLC).

### Preparation of peripheral blood mononuclear cells (PBMCs) by flow cytometry analysis

PBMCs were isolated from whole blood according to a previous report [23]. PBMCs were stained with fluorescent dye conjugated to antibodies. For pDC, anti-Human CD123-FITC (AC145) (Miltenyi Biotec., Bergisch Gladbach, Germany), BDCA4-APC (AD-17F6) (Miltenyi Biotec.), CD86-PE (IT2.2) (eBioscience, San Diego, CA, USA), and HLA-DR-PerCP (L243) (BD Bioscience, NJ, USA) were used. For mDC, anti-human Lineage Cocktail 1-FITC (Lin1) (CD3, CD14, CD16, CD19, CD20, CD56) (MφP9, NCAM16.2, 3G8, L27, SJ25C1, SK7) (BD Bioscience), CD11c-APC (MJ4-27G12) (Miltenyi Biotec.), CD1c-PE-Cy7 (L161) (BioLegend, MS, USA), CD86-PE (IT2.2) (eBioscience), and HLA-DR-PerCP (L243) (BD Bioscience) were used. CD123^+^BDCA4^+^ cells were defined as pDC and Lin1^−^CD1c^+^CD11c^+^ cells were identified as mDC. The expression levels of HLA-DR and CD86 were used as maturation markers of pDC and mDC. After staining, cells were analyzed by flow cytometry using FACS Cant II (BD Biosciences). Data were analyzed using the FlowJo software (Treestar, ON, USA).

### Measurements of incident days of influenza and URTI, and severity of symptoms

Subjects reported their conditions every day during the intervention period. The daily questionnaire asked about severity of sneeze or running nose, sore throat, cough, physical condition, fatigue, articular pain, chill, lassitude, and muscle pain. The severity of each symptom was scored using 5 grades. Evaluation of influenza and URTI incidence was performed from the severity of symptoms according to a previously reported diagnostic criterion list [25]. Measurements of severity of each symptoms were performed according to a previous report [28].

### Statistical analysis

Within group comparisons of blood and saliva samples were performed using paired *t* test. Between groups comparisons of blood and saliva samples were performed using student’s *t* test. We used Bonferoni’s method for multiple comparisons and significant *p* value was set at *p* <  0.05/3 = 0.016. Effect sizes were calculated by Cohen’s D. Cumulative incidence days of influenza and URTI symptoms, and subjective symptoms severities were compared between groups by the Chi-square test. Significant p value was set at *p* <  0.05. Data of subject’s background and training records, maturation marker on DCs, muscle damage and stress marker were reported mean ± SD. Data of cumulative day of URTI, URTI symptoms and systemic symptoms were presented as incident number (n).

## Results

### Background information and training records

Table 1 shows the background information and training records of the study subjects. No significant differences were detected in these parameters between the placebo and the LC-Plasma groups.

**Table 1:** Characteristics of subjects and training records during intervention

		Placebo Mean ± SD	LC-Plasma Mean ± SD	*p* value^a^
Characteristics of subjects				
Number of subjects		24	26	
Age	(years)	20.5 ± 0.8	20.8 ± 0.8	0.188
Height	(cm)	170.9 ± 6.5	172.2 ± 4.7	0.421
Body Weight	(kg)	60.6 ± 6.7	61.2 ± 6.8	0.740
BMI	(kg/m2)	20.7 ± 1.7	20.6 ± 1.7	0.813
Training records				
Training time	(h)	22.7 ± 7.1	21.6 ± 6.6	0.549
Physical activity	(MET-h)	175.5 ± 44.2	159.1 ± 41.2	0.188
No. of days without training	(days)	1.13 ± 1.08	1.50 ± 1.48	0.308

^a^A statistical comparison was made by student’s *t* test

### Muscle pain parameters and stress hormone

In order to evaluate the intensity of training during the intervention period, CPK, LDH, adrenaline, and cortisol were measured (Table 2). CPK, LDH, and adrenaline were significantly increased at day 14 compared to day 1 in both the placebo and LC-Plasma groups. There were no differences between the placebo and LC-Plasma groups in these indices.

**Table 2:** Markers of muscle damage and stress at Day 1 and Day 14

			Day 1	Day 14	Comparison between day 14 and day 1	Comparison between groups at day 14
			Mean ± SD	Mean ± SD	*p* value^a^ (Effect size^c^)	*p* value^b^ (Effect size^c^)
CPK	(Unit/L)	Placebo	240 ± 88	344 ± 180	0.014	0.614
					(0.587)	(0.15)
		LC-Plasma	211 ± 77	317 ± 182	0.003	
					(0.689)	
LDH	(Unit/L)	Placebo	193 ± 27	214 ± 39	0.007	0.195
					(0.634)	(0.38)
		LC-Plasma	179 ± 21	200 ± 35	0.001	
					(0.729)	
Adrenaline	(ng/mL)	Placebo	2.16 ± 0.75	6.14 ± 2.17	< 0.001	0.386
					(2.231)	(0.25)
		LC-Plasma	1.76 ± 0.81	5.67 ± 1.54	< 0.001	
					(2.806)	
Cortisol	(μg/dL)	Placebo	0.43 ± 0.22	0.43 ± 0.21	0.954	0.726
					(−0.012)	(0.10)
		LC-Plasma	0.43 ± 0.29	0.40 ± 0.26	0.672	
					(−0.084)	

^a^A statistical comparison was made by paired *t* test

^b^A statistical comparison was made by student’s *t* test

^c^Effect size was presented as Cohen’s D

### Maturation markers on DCs

**Fig. 2:**
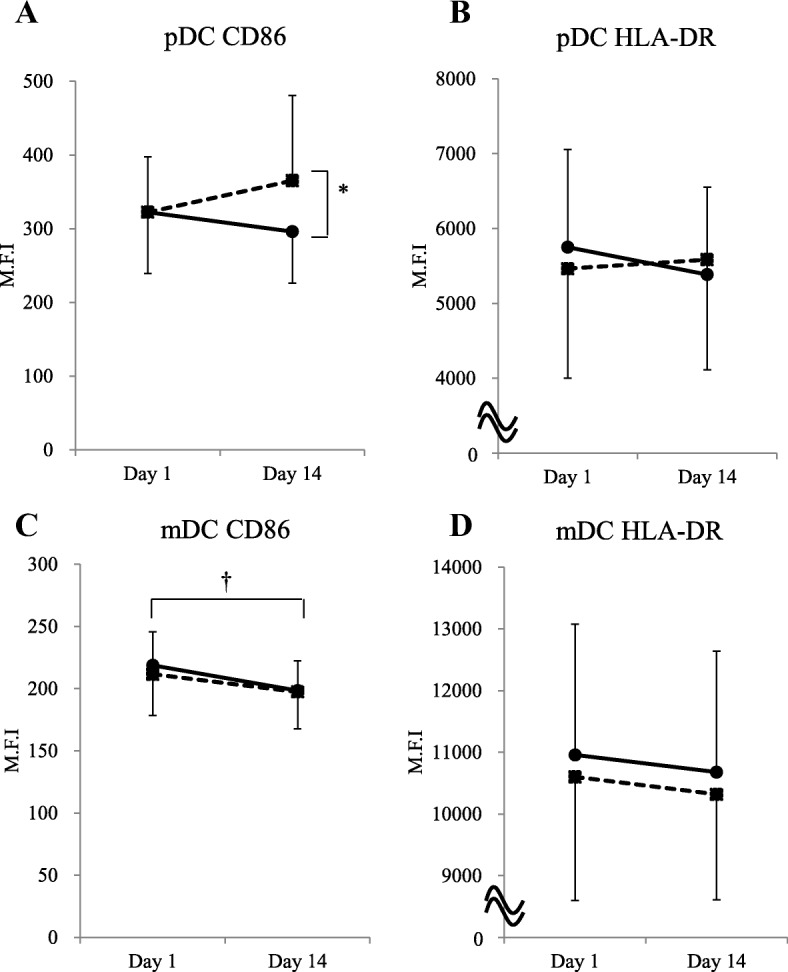
Maturation markers on DCs were measured before and after the intervention period. Comparison of (**a**) CD86 on pDC, (**b**) HLA-DR on pDC, (**c**) CD86 on mDC, (**d**) HLA-DR on mDC between placebo and LC-Plasma groups. The solid line shows the placebo group and the broken line shows the LC-Plasma group. Values are means ± SD. *: Significantly difference between groups (*p* < 0.016). Effect size was calculated by Cohen’s D as 0.72. †: Significantly difference within Placebo group (*p* < 0.016). Effect size was calculated by Cohen’s D as − 0.68

Expression of CD86 on pDC was significantly increased in the LC-Plasma group compared to the placebo group at day 14 (Fig. 2a). HLA-DR on pDC was not significantly different between groups at day 14 (Fig. 2b). CD86 and HLA-DR on mDC did not significantly change between the placebo and LC-Plasma groups at day 14 (Fig. 2c, d). A significant decrease of CD86 on mDC was detected in the placebo group after the intervention period (Fig. 2c).

### Cumulative days of influenza and URTI symptoms

The incidence of influenza and URTI symptoms was evaluated according to previous report [25]. There were no subjects judged as influenza infected. Numbers of subjects of URTI were not significantly difference between the placebo and LC-Plasma groups (Table 3). However, cumulative days of URTI positive were significantly lower in the LC-Plasma group compared to the placebo group (Table 3).

**Table 3:** Number of subjects and cumulative days of URTI during intervention period

		Symptom positive	Symptom negative	*p* value^a^
Number of subjects (n)	Placebo	13	11	0.768
	LC-Plasma	13	13	
Number of incidences (days)	Placebo	56	256	0.028
	LC-Plasma^b^	39	299	

Comparison of the cumulative number of subjects and days of URTI

^a^For each subjects and incidences were compared between the placebo and LC-Plasma groups by Chi-square test

^b^The adjusted residual of the LC-Plasma group (symptom negative) was calculated as 2.3

### Severity of subjective symptoms during the intervention period

Tables 4 and 5 shows the frequency of each evaluated symptom. The frequencies of incidence days of subjective symptoms including, sneeze or running nose, physical condition, fatigue, and articular pain were significantly lower in the LC-Plasma group than in the placebo group.

**Table 4:** Cumulative number of days of URTI symptoms during intervention period

		1, 2, 3	4, 5	*p* value^a^
Sneeze or running nose (*n*)	Placebo	52	258	0.032
(1: Severe, 2: Moderate, 3: Mild, 4: Slight, 5: Normal)	LC-Plasma^b^	36	301	
Sore throat (*n*)	Placebo	4	306	1.000
(1: Severe, 2: Moderate, 3: Mild, 4: Slight, 5: Normal)	LC-Plasma	4	333	
Cough (*n*)	Placebo	9	301	0.332
(1: Severe, 2: Moderate, 3: Mild, 4: Slight, 5: Normal)	LC-Plasma	5	332	

The cumulative number of days of URTI symptom was counted separately

^a^The cumulative days of each symptoms were compared between the placebo and LC-Plasma groups by Chi-square test

^b^The adjusted residual of the LC-Plasma group [4, 5] was calculated as 2.3

**Table 5:** Cumulative number of days of systemic symptoms during intervention period

		1, 2, 3	4, 5	*p* value^a^
Physical condition (*n*)	Placebo	184	125	0.030
(1: Very bad, 2: Bad, 3: Normal, 4: Good, 5: Very good)	LC-Plasma^b^	171	166	
Fatigue (*n*)	Placebo	128	182	0.032
(1: Severe, 2: Moderate, 3: Mild, 4: Slight, 5: Normal)	LC-Plasma^c^	110	225	
Articular pain (*n*)	Placebo	16	294	0.016
(1: Severe, 2: Moderate, 3: Mild, 4: Slight, 5: Normal)	LC-Plasma^d^	5	332	
Chill (*n*)	Placebo	2	308	0.516
(1: Severe, 2: Moderate, 3: Mild, 4: Slight, 5: Normal)	LC-Plasma	5	332	
Lassitude (*n*)	Placebo	52	258	0.098
(1: Severe, 2: Moderate, 3: Mild, 4: Slight, 5: Normal)	LC-Plasma	75	262	
Muscle pain (*n*)	Placebo	62	248	1.000
(1: Severe, 2: Moderate, 3: Mild, 4: Slight, 5: Normal)	LC-Plasma	68	268	

The cumulative number of days of each symptom was counted separately

^a^The cumulative days of each symptoms were compared between the placebo and LC-Plasma groups by Chi-square test

^b^The adjusted residual of the LC-Plasma group [4, 5] was calculated as 2.2

^c^The adjusted residual of the LC-Plasma group [4, 5] was calculated as 2.2

^d^The adjusted residual of the LC-Plasma group [4, 5] was calculated as 2.6

## Discussion

LC-Plasma is a unique LAB which activates plasmacytoid dendritic cells (pDC) in healthy subjects. However, efficacy during consecutive HIE have not been evaluated. We conducted human study and researched the effects of LC-Plasma in athletes. The results indicated that LC-Plasma supplementation for 13 days would able to increase maturation marker of pDC (CD86) and decrease cumulative days of URTI symptom. Furthermore, we observed that LC-Plasma ingestion could decrease cumulative days of fatigue. These findings indicated that intake of LC-Plasma would prevent URTI infection via pDC activation and improve fatigue accumulation, suggesting that our hypothesis was acceptable.

There were no significantly differences between the groups in the training records during intervention (Table 1). It was reported that exercise for more than 11 h a week is reported to be high intensity and increasing URTI morbidity [29]. Although we did not strictly control exercise, both groups exercised for more than 11 h a week. Therefore, we thought that high intensity exercise was loaded to both groups as the physical condition of subjects became decrease. In addition, CPK and LDH as markers of muscle damage, and adrenaline and cortisol as stress hormones were also no different at day 14 (Table 2). These results suggested that exercise stress load between the placebo and LC-Plasma group was comparable in this study.

CPK, LDH and adrenaline were significantly increased at day 14 compared to day 1. Our data showed that exercise during the intervention period was high enough to affect markers of muscle damage and stress hormone. CPK and adrenaline reported to increase after HIE and to be involved in immunity and fatigue [30–32]. We found that maturation markers (CD86 and HLA-DR) on DCs were decreased in the placebo group (Fig. 2). These results indicated that DCs are also involved in immuno-reduction under high stress condition such as consecutive HIE.

We showed that CD86 on pDC increased in the LC-Plasma group compared to the placebo group after consecutive HIE. This suggests that LC-Plasma increases pDC maturation in athletes under HIE as in general healthy subjects as reported previously [23].

Cumulative incidence days of URTI and symptom like sneeze or running nose decreased in the LC-Plasma group. Since pDC were reported to play antiviral functions [10] and that 70% of the causes of URTI were viral infections [12], we can conclude that LC-Plasma supplementation relieves URTI morbidity during HIE via pDC activation. In previous reports, LC-Plasma supplementation for more than 4 weeks was effective in general healthy subjects [23, 24, 28]. We found that LC-Plasma was effective even in a short intervention period of 13 days. Because this study was conducted under high stress conditions, the effect of LC-Plasma supplementation could be confirmed earlier and clearly.

We found that fatigue accumulation was lower in the LC-Plasma group compared to the placebo group. There are no reports regarding probiotics improving fatigue in athletes. Therefore, here we showed for the first time that LAB material such as LC-Plasma supplementation was effective for improving fatigue during consecutive HIE. For people under physically stressful conditions such as athletes, fatigue accumulation is a serious problem [33–35], therefore our finding is valuable for athletes.

Since there was no significant difference in markers of muscle damage and stress in LC-Plasma group compared to Placebo, LC-Plasma might not directly affect recovery through these markers. Although detailed mechanism for fatigue reduction by LC-Plasma is not fully understood, one possibility is autonomic nerves, affected by HIE and related to fatigue [36]. Athletes who develop chronic fatigue or depression due to excessive HIE were reported to have abnormal autonomic nerves [37]. It was also reported that TLR9 knock out mice exhibited changes of autonomic nerve functions (heart rate and pulse interval) and behavior with responsiveness to stress [38]. Considering that LC-Plasma is a ligand of TLR9 [22], LC-Plasma might affect autonomic nerves and accordingly improve fatigue state via TLR9. Further research is necessary in order to confirm the precise mechanism of LC-Plasma regarding fatigue.

There were some limitations regarding the analysis, which may affect the results of this study. The study subjects were composed of only male and university students. Furthermore, even though the exercise load was not different between groups, the training regime was not strictly controlled. Since this study was conducted in 13 days of ingestion which is thought to be relatively short period, the efficacy of LC-Plasma for athletes during long-term ingestion was unknown. More extensive studies with a wide range of subjects or homogeneous exercise load like ergometer exercise or long and high intensive exercise are required to confirm our findings.

## Conclusions

In this study, we showed that heat-killed LC-Plasma supplementation relieves URTI morbidity and symptoms during consecutive HIE via activation of pDC. Moreover, LC-Plasma ingestion decreased fatigue accumulation. We suggest that heat-killed LC-Plasma was beneficial to solve problems of athletes such as immune-reduction and increased fatigue.
